# Diversity and Inclusion in Libraries: A Call to Action and Strategies for Success

**DOI:** 10.5195/jmla.2020.1027

**Published:** 2020-10-01

**Authors:** Lisa Liang Philpotts

**Affiliations:** 1 lphilpotts@mgh.harvard.edu, Treadwell Library, Massachusetts General Hospital, Boston, MA

Medical librarians will immediately recognize the names Shannon D. Jones, AHIP, and Beverly Murphy, AHIP, FMLA. Both are leaders in the profession who have facilitated numerous conversations on diversity and inclusion, and have contributed to multiple presentations and panels on a national level. Notably, in 2018 Murphy became the Medical Library Association's first African American president. The two have teamed up to serve as editors of *Diversity and Inclusion in Libraries: A Call to Action and Strategies for Success*, a timely, much needed book with chapters written by thirty different contributors.

The contributors largely write from the perspective of librarians in academic settings, though there are also narratives from public librarians and librarians who have led initiatives in professional organizations. As a hospital librarian, I found that the book gave me plenty of ideas that I could implement in my workplace. A distinguishing feature of the book is that it not only presents the history and research on diversity in librarianship, but also provides numerous real-life examples and practical recommendations for supporting diversity in libraries.

*Diversity and Inclusion in Libraries* focuses on librarianship in the United States, and, thus, much of content necessarily focuses on addressing the lack of racial diversity in the profession. As pointed out by multiple contributors, the American Library Association's *Diversity Counts* study reported that 88% of credentialed librarians in the workforce are White [1], and readers will find multiple suggestions of how to recruit, support, and retain librarians of color throughout *Diversity and Inclusion in Libraries*. Other aspects of diversity are addressed in chapters about support for transgender and gender nonconforming students, disability and the library workplace, support for immigrant families, and the experiences of Black male and first-generation college student librarians.

Busy librarians will be pleased to find that the chapters are informative and succinct, with references and contact information for the contributors included for those who want to seek out more detail. The volume is organized into three parts. Part I: “Why Diversity and Inclusion Matter” provides necessary background information for the reader, including overviews of notable African American librarians, a summary of trends and themes in diversity-focused library science literature, and an overview of the demographics and disparities in the librarian workforce. Part I also includes primers on implicit bias, microaggressions, and subversive librarianship.

Part II: “Equipping the Library Staff” includes case studies and a slew of practical recommendations for readers who wish to promote diversity and inclusion in their institutions. Those interested in the specifics of what works and what missteps to avoid when trying to implement diversity initiatives will find this part valuable.

Part III: “Voices from the Field” spotlights the lived experiences of library staff in their own words. Most important in diversity and inclusion initiatives is listening to those who have experienced oppression, and these contributors have generously shared the barriers they have encountered and the support they have received while working in libraries for the benefit of all.

One issue in this otherwise excellent volume is that the term “transgendered” is used a few times. Organizations like the National LGBTQIA+ Health Education Center consider this term outdated and insensitive and suggest using “transgender” instead [2]. Aside from that, this book is highly recommended for librarians in all settings who seek to promote diversity and inclusion in their workplaces and profession. It also has potential as a text for library science faculty and students who seek to understand diversity issues in the profession, and many of the chapters could serve as great conversation starters. In short, this book can empower us all to take steps to address inequities and better support each other and our patrons.
